# Ten-Year Surgical Outcomes and Prognostic Factors for French-Door Laminoplasty in the Treatment of Multilevel Cervical Spondylotic Myelopathy

**DOI:** 10.1155/2020/3627071

**Published:** 2020-05-06

**Authors:** Guoliang Chen, Xizhe Liu, Ningning Chen, Bailing Chen, Xuenong Zou, Fuxin Wei, Shaoyu Liu

**Affiliations:** ^1^Department of Orthopedic Surgery, The Seventh Affiliated Hospital, Sun Yat-sen University, Shenzhen, China; ^2^Department of Spine Surgery, Guangdong Provincial Key Laboratory of Orthopaedics and Traumatology/Orthopaedic Research Institute, The First Affiliated Hospital, Sun Yat-sen University, Guangzhou, China

## Abstract

**Objective:**

To analyze the ten-year surgical outcomes and postoperative complications of French-Door laminoplasty (FDL) in the management of multilevel cervical spondylotic myelopathy (MCSM) and analyze the prognostic factors for FDL in treating MCSM.

**Methods:**

64 patients with MCSM, who were operated by FDL, were included in this study and followed up for at least 10 years. Clinical assessments including modified Japanese Orthopaedic Association (mJOA) score, age at surgery, preoperative symptom duration, operative time, blood loss and postoperative complications, radiological assessments including Cobb angle, cervical range of motion (ROM), intramedullary signal intensity on T2W MRI, canal narrowing ratio (CNR), and maximum spinal cord compression (MSCC). mJOA score, Cobb angle, cervical ROM, intramedullary signal intensity on T2W MRI, and CNR were assessed before surgery and at the final follow-up.

**Results:**

The average mJOA score was significantly improved from preoperative 10.32 ± 1.63 points to 15.10 ± 0.62 points at the final follow-up (*p* < 0.05). The average RR of the mJOA score at the final follow-up was 69.10 ± 7.32%. The cervical Cobb angle and ROM decreased significantly at the final follow-up. Patients with high intramedullary signal intensity of T2W MRI or CNR more than 50% showed a lower RR of the mJOA score. Correlation analysis revealed that preoperative symptom duration and intramedullary signal intensity of T2W MRI, CNR, MSCC, and blood loss were significantly correlated with the RR of the mJOA score. Gender, operative method, and age at surgery were significantly correlated with the preservation rate of ROM. Operative time was significantly correlated with the incidence of axial symptoms.

**Conclusions:**

The ten-year clinical outcomes of FDL were satisfactory. Higher intramedullary signal intensity of T2W MRI and a greater CNR predicted poorer prognoses.

## 1. Introduction

Cervical spondylotic myelopathy (CSM) is a clinical condition manifested with symptoms caused by spinal cord compression owing to disc herniation, ligament hypertrophy, or the presence of osteophytes [1]. For patients failing to respond to conservative treatment, surgically decompressing the spinal cord and thus alleviating the neurological deterioration are imperative [2]. For the multilevel cervical spondylotic myelopathy (MCSM), the anterior approach surgery is not preferred due to its high complication rate [3]. Posterior approaches such as laminoplasty and laminectomy have been recognized as the effective methods in treating MCSM [4–6]. However, according to previously published studies, operation-related complications, such as cervical kyphosis, segmental instability, and neurological deterioration which were likely due to cervical instability after posterior structures being removed, were not uncommon in patients treated by laminectomy [7]. Therefore, laminoplasty is put into application as an alternative to laminectomy, which permits adequate decompression while maintaining mechanical stability and motion of the cervical spine [8]. French-Door laminoplasty (FDL), introduced by Kurokawa et al. [9], involves opening the “door” in the midline, which creates a symmetrical expansion of the canal. The advantages of FDL are to expand the spinal canal and preserve the posterior structures of the cervical spine [10]. Though numerous studies have reported the short- and midterm surgical results of FDL are definite, there were also several postoperative complications of FDL such as C5 palsy, axial symptoms, decrease of the cervical range of motion (ROM) or Cobb angle, and reclosure of the lamina [11]. However, there are insufficient long-term follow-up studies of verifying the surgical efficacy and factors affecting its efficacy. In order to study the long-term surgical outcomes, especially the postoperative neurological function, cervical ROM and alignment, complications, and prognostic factors of FDL for treating MCSM, we carried out this retrospective study.

## 2. Materials and Methods

### 2.1. Study Population

From March 2007 to March 2009, a total of 76 MCSM patients received cervical laminoplasty performed by the same chief surgeon in the First Affiliated Hospital, Sun Yat-sen University. Patients with a history of trauma, infection, tumor, or previous cervical surgery were excluded from this study. Sixty-four patients (46 males, 18 females) who were followed for at least 10 years were included in this study. This study was approved by the ethics committee of our hospital, and written informed consent from each patient was obtained.

### 2.2. Surgical Techniques

The FDL was performed in compliance with Kurokawa's^9^ method with some modifications. After detaching bilateral paravertebral muscles from the spinous processes, all spinous processes within the surgical range were removed. The center of the laminae was cut using a fretsaw. Bilateral gutters were created as hinges by a rongeur at the border of the laminae and facet. After halves of the laminae were elevated, a sizeable hydroxyapatite spacer was tied to bridge the bilateral edges of the laminae and fixed with wires. After laminoplasty, all patients' C2 semispinalis were sutured in situ, followed by putting the drainage tube and closing wound in layers in the end.

### 2.3. Evaluations

#### 2.3.1. Clinical Assessments

The age at surgery, preoperative symptom duration, operative time, and blood loss were recorded, and postoperative complications such as incision infection, axial symptoms, and C5 palsy were also recorded at each follow-up.

Neurological function was evaluated using the modified Japanese Orthopaedic Association (mJOA) scoring system before surgery and at each follow-up. The recovery rate (RR) of the mJOA score (%) was calculated using the following formula.

(1) $$\begin{array}{c} \text{RR of JOA score}=\frac{\text{postoperative mJOA score}–\text{preoperative mJOA score}}{17–\text{preoperative mJOA score }}\times 100\%. \end{array}$$

#### 2.3.2. Radiological Measurements

**Figure 1:**
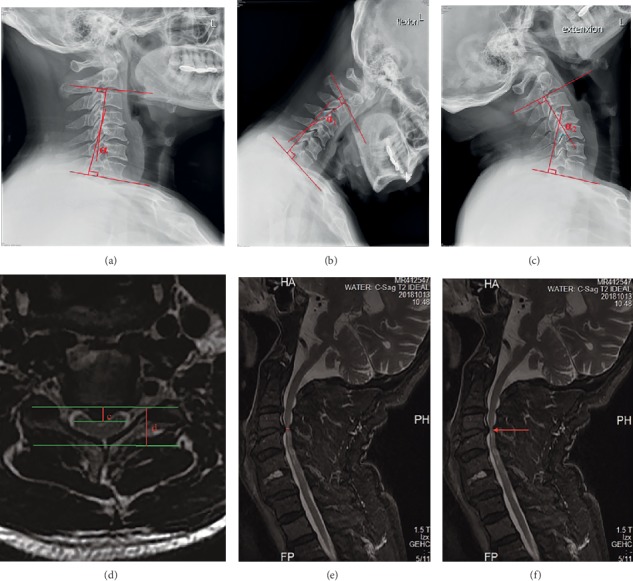
Radiological measurements: (a) the measurement of Cobb angle on the neutral position lateral X-ray; (b, c) the measurement of cervical range of motion (ROM) on the extension and flexion lateral X-ray; (d) the measurement of canal narrowing ratio (CNR) on traverse T2W MRI: CNR = *c*/*d*; (e) the measurement of maximum spinal cord compression on midsagittal T2W MRI; (f) the diagram of intramedullary signal of T2W MRI: the red arrow indicates high signal.

Anteroposterior, lateral, and extension-flexion radiographs; cervical CT; and MRI were conducted before surgery and at each follow-up. The Cobb angle which represents the cervical alignment and cervical ROM were measured using lateral radiographs and dynamic lateral radiographs, respectively. The intramedullary signal intensity, the canal narrowing ratio (CNR), and the maximum spinal cord compression (MSCC) on T2W MRI of all patients were recorded before surgery. All patients' imaging was assessed by the same three researchers independently and repeated for three times (Figure 1).

#### 2.3.3. Statistics

Results were statistically analyzed using SPSS 23.0 software (SPSS, Chicago, IL, USA). Data was expressed as the mean ± standard deviation. A paired *t*-test was used to compare the mJOA score before and after surgery. The correlation between continuous variables and clinical outcomes was analyzed using the Pearson correlation analysis. The correlation between classification variables and clinical outcomes, preoperative factors, and postoperative complications was analyzed using the Spearman correlation analysis. An independent samples *t*-test was used to compare the postoperative results between subgroups. *p* value < 0.05 indicated a significant difference.

## 3. Results and Discussion

### 3.1. Results

#### 3.1.1. Baseline Data

The mean preoperative symptom duration was 31.80 ± 52.42 months, the mean age at surgery was 55.60 ± 13.51 years, the mean operative time was 151.32 ± 32.30 min, and the mean blood loss was 152.45 ± 93.56 ml (Table 1).

#### 3.1.2. Neurological Function

The average mJOA score was significantly improved from preoperative 10.32 ± 1.63 points to 15.10 ± 0.62 points at the final follow-up (*p* < 0.05). The average RR of the mJOA score at the final follow-up was 69.10 ± 7.32% (Table 2).

### 3.2. Radiographic Outcomes

The mean Cobb angle reduced significantly from preoperative 16.10 ± 8.09° to 11.79 ± 6.52° at the last follow-up (*p* < 0.05); the mean loss rate of the Cobb angle was 26.90 ± 12.12%. The cervical ROM at the final follow-up decreased significantly from preoperative 44.24 ± 13.18° to 26.65 ± 7.61°; the preservation rate of ROM was 59.10 ± 10.84% (Table 2).

### 3.3. Postoperative Complications

One case of superficial infection, two cases of axial symptoms, and one case of C5 palsy were recorded. No other complications such as cervical kyphosis, internal failure, and reclosure of the lamina were observed. All the superficial infections were cured after oral antibiotics were given for two weeks, except one case which was performed with debridement and resutured. All the patients diagnosed with axial symptoms and C5 palsy recovered spontaneously within 6 months after surgery.

### 3.4. Correlation Analysis

The results of correlation analysis showed that the preoperative duration of symptom, CNR, MSCC, and intramedullary signal of T2W MRI were significantly correlated with the RR of the mJOA score, and the gender was significantly correlated with the postoperative ROM preservation rate. However, none of the investigated variables were proven to be significantly correlated with the change of the Cobb angle (Table 3).

The results of correlation analysis between the included factors and postoperative complications demonstrated that operating time was significantly associated with axial symptoms, gender was significantly associated with C5 palsy, and none of all the factors were significantly associated with incision infection (Table 4).

### 3.5. Subgroup Comparisons

Based on the results of correlation analysis, all patients were further divided into different groups, respectively, according to gender (male or female), age at surgery (≥60 y or <60 y), preoperative intramedullary signal intensity of T2W MRI (high signal or equal and low signal), and the CNR (≥50% or <50%). Then the postoperative RR of mJOA, loss rate of Cobb angle, and preservation rate of cervical ROM were compared.

At the last follow-up, patients with high intramedullary signal intensity of T2W MRI before surgery have a lower RR of the mJOA score than those who showed equal or low signal before operation. The RR of the mJOA score of patients with greater CNR (≥50%) was significantly lower than that of patients with lower CNR (<50%). The gender and age at surgery did not influence the postoperative RR of the mJOA score. Male patients' postoperative ROM preservation rate was significantly higher than that of female patients. For patients of different gender, age at surgery, preoperative intramedullary signal of T2W MRI, and CNR, no significant difference was observed in terms of postoperative Cobb angle change (Table 5).

## 4. Discussion

In this study, 64 patients with MCSM who underwent FDL were included. By analyzing the clinical and radiological results, incidence of complications, and prognostic factors, we found that FDL achieved satisfactory neurological outcomes, the preoperative duration of symptom, CNR, MSCC, and intramedullary signal of T2W MRI were significantly correlated with the RR of the JOA score, gender was significantly correlated with the postoperative ROM preservation rate, and patients with high intramedullary signal intensity of T2W MRI or greater CNR (≥50%) showed a lower RR of the mJOA score.

Current studies demonstrated that the main pathogenesis of spinal cord impairment in CSM was the chronic compression and the ischemia and neuroinflammation secondary to compression [12, 13]. The surgical procedures might achieve improvement of neurological function by decompressing and increasing the perfusion of spinal cord [14]. To our knowledge, laminoplasty which decompresses the spinal cord by enlarging the spinal canal has been recognized as one of the most important surgical practices to treat MCSM and achieved satisfactory short- and midterm clinical outcomes [15]. Besides, compared with laminectomy and fixation, previous studies have confirmed that laminoplasty not only achieved similar neurological improvement and better preservation of cervical ROM but also decreased the complication rate significantly [6, 8]. In this study, the average RR of the mJOA score at 10 years after surgery was 69.10 ± 7.32%, and overall complication rate was relatively low (5/64), which were in accordance with that of previous studies [6, 15, 16].

The correlation between preoperative risk factors and postoperative outcomes is still controversial. Our results revealed that preoperative symptom duration, CNR, MSCC, intramedullary signal intensity of T2W MRI, and blood loss were significantly negatively correlated with the RR of the mJOA score. In the above factors, the preoperative symptom duration, CNR, and MSCC represent the degree of compression while the intramedullary signal intensity of T2W MRI represents the degree of impairment. It has been reported that the spinal cord compression may be the initiator of spinal impairment of CSM [13]. A previous study confirmed that the sensitivity and specificity of spinal cord compression in CSM were 100% and 79.6%, respectively [17]. The larger the CNR, the lesser the MSCC means the greater compression, and the long-term chronic compression would disturb the blood supply of the spinal cord, which might lead to the pathological changes, such as parenchymal edema and ischemic and cystic degeneration of the spinal cord. This could explain why the CNR and MSCC were significantly negatively correlated with the RR of the mJOA score. In addition, the pathological changes listed above, which are considered to be the beginning of irreversible damage to the spinal cord, could be reflected on T2W MRI as high signal intensity (HSI) [18]. This might provide information for us to evaluate the prognosis of the patients with CSM preoperatively.

The relationship between T2W MRI HSI and neurological function in CSM has also been a research hotspot [19]. Many studies reported that patients without high intramedullary signal of T2W MRI before surgery achieved better outcomes, and they deduced that the preoperative intramedullary signal intensity of T2W MRI was a valuable prognostic factor [20, 21]. Previous studies found that patients presenting with preoperative HSI in T2W MRI had longer duration of symptoms, more severe symptoms, and a higher risk of rapid progression [18, 22]. In this study, the clinical efficacy of patients with high intramedullary signal of T2W MRI was poorer, resulting in similar findings. Moreover, the RR of mJOA has a significantly negative correlation with the blood loss. It might be due to limited blood supply of the spinal cord during surgery and lead to further impairment and poor clinical outcome.

One of the purposes of cervical laminoplasty is to preserve cervical ROM and maintain the cervical stability and lordosis after decompression [23]. However, some studies showed that postoperative cervical ROM decreased about 30%-70% after laminoplasty [24]. There are various studies focusing on the factors affecting the Cobb angle and ROM after laminoplasty, but the specific reasons remained unclear. Several surgeons considered that the posterior cervical muscles played an important role in the change of Cobb angle and cervical ROM after operation. Fujimura and Nishi [25] found that the area of posterior cervical muscles by CT cross-sectional scan was correlated with the changing curve of ROM. And a variety of modified methods that protect the posterior cervical muscles, such as separating the unilateral paravertebral muscles or preserving C2 semispinalis, have been used and achieved better outcomes of ROM [26]. The surgical approach adopted in this study requires separation of bilateral paravertebral muscles and C2 semispinalis, but C2 semispinalis was sutured in situ after decompression. In this study, the average preservation rate of the ROM was 59.06%, the mean loss of the Cobb angle was 4.16 ± 2.62°, the result of correlation analysis revealed male and young patients achieved better preservation rate of ROM after surgery, and we considered that male and young patients might follow more rigorous rehabilitation exercises after surgery.

It has been reported that the complications following laminoplasty, such as axial symptoms and C5 palsy, resulted in serious impact on a patient's quality of life postoperatively [27–29]. Studies asserted that the incidence of axial symptoms after laminoplasty was up to 45%-80% and the overall incidence of C5 palsy was about 4.6% [30]. Current studies believed that factors such as the destruction of the posterior cervical structures, the instability of the facet joint, and the atrophy of the posterior cervical muscles caused by prolonged intraoperative traction were correlated with the axial symptoms [31] and that C5 palsy is correlated to traction after the spinal cord drifted backward, segmental ischemia, and ischemia-reperfusion injury of the spinal cord [30–32]. In our study, long operative time was one of the risk factors correlated with the axial symptoms. This might be due to prolonging the muscle traction during operation, which induced the injury of posterior cervical muscles. Based on the clinical results of satisfactory long-term neurological improvement and relatively low complication rates in this study, we hold the opinion that FDL was an effective method to treat MCSM, but whether the effectiveness of FDL is superior to other forms of laminoplasty, a further comparative study is imperative.

In this study, there are several limitations worth noting. Relatively small sample size may result in low credibility. The information bias is easy to occur due to the subjectivity of the mJOA score. Besides, the study about cervical ROM is not comprehensive because we have not measured the ROM of cervical lateroversion and rotation. In order to obtain a precise conclusion, a prospective study with large samples, long-term follow-up, and more evaluation indexes is necessary.

## 5. Conclusions

FDL is effective for treating MCSM, and the ten-year surgical results were satisfactory. Long preoperative symptom duration, high intramedullary signal of T2W MRI, and a greater CNR (≥50%) indicate a poor neurological recovery. Male and young patients achieved a better preservation rate of the ROM after surgery.

## Figures and Tables

**Table 1:** Baseline data of the patients.

Indictor	Result
Number of patients	64
Male	46
Female	18
Age at surgery (year)	55.60 ± 13.51
Symptom duration (month)	31.80 ± 52.42
Operative time (min)	151.32 ± 32.30
Blood loss (ml)	152.45 ± 93.56

**Table 2:** Neurological function and radiographic data.

Indicator	Result
*mJOA score*	
Preoperative	10.32 ± 1.63
Final follow-up	15.10 ± 0.62^∗^
Recovery rate of mJOA score at the final follow-up (%)	69.10 ± 7.32
*Radiographic data*	
Preoperative Cobb angle (°)	16.10 ± 8.09
Cobb angle at the final follow-up (°)	11.79 ± 6.52^∗^
Loss rate of Cobb angle at the final follow-up (%)	26.90 ± 12.12
Preoperative cervical range of motion (°)	44.24 ± 13.18
Cervical range of motion at the final follow-up (°)	26.65 ± 7.61^∗^
Preservation rate of cervical range of motion at the final follow-up (%)	59.10 ± 10.84

^∗^Compared with preoperative, *p* < 0.05.

**Table 3:** The results of correlation analysis between preoperative factors, neurological function, and radiological outcomes.

Preoperative factors	The recovery rate of mJOA score (%)		The loss rate of cobb angle (%)		The preservation rate of range of motion (%)	
	Correlation coefficient	*p*	Correlation coefficient	*p*	Correlation coefficient	*p*
Gender	-0.109	0.390	0.003	0.938	-0.369	0.002^∗^
Age at surgery	-0.025	0.845	-0.129	0.320	-0.276	0.035^∗^
Symptom duration	-0.298	0.022^∗^	0.097	0.495	-0.058	0.698
Operative time	-0.045	0.687	-0.023	0.967	0.021	0.843
Blood loss	-0.401	0.001^∗^	-0.254	0.067	-0.020	0.889
Cobb angle	0.016	0.913	-0.126	0.363	0.087	0.510
Cervical range of motion	0.013	0.923	-0.005	0.976	-0.372	0.004^∗^
Intramedullar signal of T2W MRI	-0.562	0.000^∗^	-0.128	0.318	0.191	0.127
Canal narrowing ratio	-0.418	0.000^∗^	-0.039	0.751	0.229	0.067
Maximum spinal cord compression	0.251	0.045^∗^	0.129	0.269	-0.196	0.133

^∗^ *p* < 0.05.

**Table 4:** The results of correlation analysis between preoperative factors and postoperative complications.

Preoperative factors	Incision infection		Axial symptoms		C5 palsy	
	Correlation coefficient	*p*	Correlation coefficient	*p*	Correlation coefficient	*p*
Gender	-0.143	0.260	-0.060	0.618	0.266	0.026^∗^
Age at surgery	0.027	0.829	-0.020	0.892	0.140	0.234
Symptom duration	0.011	0.915	0.053	0.691	0.011	0.993
Operative time	0.060	0.650	0.249	0.046^∗^	-0.024	0.852
Blood loss	-0.020	0.869	0.047	0.719	0.003	0.974
mJOA score	-0.059	0.616	-0.200	0.133	0.158	0.235
Cobb angle	-0.042	0.749	0.159	0.203	-0.022	0.847
Cervical range of motion	0.065	0.659	-0.073	0.562	0.038	0.798
Intramedullar signal of T2W MRI	-0.066	0.600	-0.050	0.705	0.005	0.957
Canal narrowing ratio	-0.046	0.705	-0.021	0.873	-0.165	0.221
Maximum spinal cord compression	0.132	0.336	0.089	0.455	0.234	0.077

^∗^ *p* < 0.05.

**Table 5:** The clinical and radiographic outcomes at the last follow-up based on gender, age, surgical method, and preoperative intramedullar signal of T2W MRI.

Case		The recovery rate of mJOA score (%)		The loss rate of Cobb angle (%)		The preservation rate of range of motion (%)	
		Mean value	*p*	Mean value	*p*	Mean value	*p*
Gender							
Male	46	70.34 ± 8.79	0.375	27.01 ± 12.79	0.979	65.69 ± 10.51	0.003^∗^
Female	18	69.06 ± 5.72		27.30 ± 13.22		55.43 ± 13.325	
Age at surgery							
<60 y	38	69.55 ± 8.71	0.976	28.53 ± 14.52	0.589	61.14 ± 11.23	0.325
≥60 y	26	71.06 ± 7.07		25.08 ± 10.31		65.50 ± 12.10	
Preoperative intramedullar signal of T2W MRI							
High	34	66.78 ± 8.50	0.000^∗^	24.10 ± 9.15	0.314	65.28 ± 10.34	0.125
Equal or low	30	73.58 ± 5.42		29.39 ± 15.19		60.42 ± 13.71	
Canal narrowing ratio							
≥50%	27	66.25 ± 8.45	0.001^∗^	26.03 ± 12.52	0.745	65.76 ± 12.41	0.067
<50%	38	72.90 ± 6.01		28.02 ± 13.44		60.32 ± 11.85	

^∗^ *p* < 0.05.

## Data Availability

The data used to support the findings of this study were collected by authors under license and so cannot be made freely available. Requests for access to these data should be made to the corresponding authors.
